# A New Single Nucleotide Polymorphism Database for Rainbow Trout Generated Through Whole Genome Resequencing

**DOI:** 10.3389/fgene.2018.00147

**Published:** 2018-04-24

**Authors:** Guangtu Gao, Torfinn Nome, Devon E. Pearse, Thomas Moen, Kerry A. Naish, Gary H. Thorgaard, Sigbjørn Lien, Yniv Palti

**Affiliations:** ^1^National Center for Cool and Cold Water Aquaculture, ARS-USDA, Kearneysville, WV, United States; ^2^Department of Animal and Aquacultural Sciences, Faculty of Biosciences, Centre of Integrative Genetics, Norwegian University of Life Sciences, Ås, Norway; ^3^Fisheries Ecology Division, Southwest Fisheries Science Center, National Marine Fisheries Service, Santa Cruz, CA, United States; ^4^AquaGen, Trondheim, Norway; ^5^School of Aquatic and Fishery Sciences, University of Washington, Seattle, WA, United States; ^6^School of Biological Sciences and Center for Reproductive Biology, Washington State University, Pullman, WA, United States

**Keywords:** rainbow trout, SNP discovery, genome resequencing, doubled haploid, paralogous sequence variants

## Abstract

Single-nucleotide polymorphisms (SNPs) are highly abundant markers, which are broadly distributed in animal genomes. For rainbow trout (*Oncorhynchus mykiss*), SNP discovery has been previously done through sequencing of restriction-site associated DNA (RAD) libraries, reduced representation libraries (RRL) and RNA sequencing. Recently we have performed high coverage whole genome resequencing with 61 unrelated samples, representing a wide range of rainbow trout and steelhead populations, with 49 new samples added to 12 aquaculture samples from AquaGen (Norway) that we previously used for SNP discovery. Of the 49 new samples, 11 were double-haploid lines from Washington State University (WSU) and 38 represented wild and hatchery populations from a wide range of geographic distribution and with divergent migratory phenotypes. We then mapped the sequences to the new rainbow trout reference genome assembly (GCA_002163495.1) which is based on the Swanson YY doubled haploid line. Variant calling was conducted with *FreeBayes* and *SAMtools mpileup*, followed by filtering of SNPs based on quality score, sequence complexity, read depth on the locus, and number of genotyped samples. Results from the two variant calling programs were compared and genotypes of the double haploid samples were used for detecting and filtering putative paralogous sequence variants (PSVs) and multi-sequence variants (MSVs). Overall, 30,302,087 SNPs were identified on the rainbow trout genome 29 chromosomes and 1,139,018 on unplaced scaffolds, with 4,042,723 SNPs having high minor allele frequency (MAF > 0.25). The average SNP density on the chromosomes was one SNP per 64 bp, or 15.6 SNPs per 1 kb. Results from the phylogenetic analysis that we conducted indicate that the SNP markers contain enough population-specific polymorphisms for recovering population relationships despite the small sample size used. Intra-Population polymorphism assessment revealed high level of polymorphism and heterozygosity within each population. We also provide functional annotation based on the genome position of each SNP and evaluate the use of clonal lines for filtering of PSVs and MSVs. These SNPs form a new database, which provides an important resource for a new high density SNP array design and for other SNP genotyping platforms used for genetic and genomics studies of this iconic salmonid fish species.

## Introduction

The rainbow trout (*Oncorhynchus mykiss*) is an iconic salmonid fish species with a remarkably diverse life history, and has a wide interest as a model research organism as well as high economic value for the sport fishing and aquaculture industries. The substantial scientific and economic interests in this species justify the continued development of genomic resources. Much effort has been devoted in recent years for developing genomic resources for research in rainbow trout, including a draft genome assembly (Berthelot et al., 2014), a high-density 57K SNP array (Palti et al., 2015a), a dense genetic linkage map (Gonzalez-Pena et al., 2016), and recently the annotated reference genome sequence (GenBank assembly Accession GCA_002163495, RefSeq assembly accession GCF_002163495).

Whole genome association analyses (WGA) can be useful for dissecting complex traits in natural populations and in aquaculture breeding programs and a lot of research in rainbow trout has been using WGA for better understanding of the genetics underlying complex traits (Hohenlohe et al., 2011; Miller et al., 2012; Pearse et al., 2014; Gonzalez-Pena et al., 2016; Vallejo et al., 2016, 2017a,b; Leitwein et al., 2017). One of the major limitations for WGA in rainbow trout has been the lack of dense sets of genetic markers to provide adequate coverage of the chromosomes. Early efforts used targeted single-gene sequencing to discover and characterize a restricted number of Single-nucleotide polymorphisms (SNPs) (AbadÍA-Cardoso et al., 2011). Another approach has been to use genotyping by sequencing methods such as restriction site associated DNA (RAD) markers (e.g., Miller et al., 2012; Palti et al., 2015b), but there have been many technical difficulties in comparing and transferring results across studies. More recently, a 57K SNP array was developed and has significantly improved our ability to conduct whole genome studies in rainbow trout (Gonzalez-Pena et al., 2016; Vallejo et al., 2016, 2017a,b; Yoshida et al., 2017; Larson et al., 2018). However, the chromosome sequences that we have recently released in GenBank (GCA_002163495.1), generated a great opportunity for large-scale SNP discovery using whole genome resequencing of target populations. A comprehensive SNP database from genome resequencing data can further improve the design and selection of a new SNP arrays by improving the genome coverage and spacing of the SNPs as well as selecting markers for follow up studies within targeted regions of the genome and for particular populations.

All ray-finned fish share an additional (3R) round of ancestral genome duplication in their evolutionary history compared to mammals and birds, but the salmonids underwent a further salmonid-specific (Ss4R) whole genome duplication (WGD) event ~95 MYA (Lien et al., 2011, 2016; Berthelot et al., 2014; Macqueen and Johnston, 2014). Despite the long time that have passed since the Ss4R WGD, large regions of salmonid genomes still behave in a tetraploid manner in extant species (Allendorf and Thorgaard, 1984; Lien et al., 2011, 2016; Allendorf et al., 2015; Waples et al., 2016). A paralogous sequence variant (PSV) is a variant in a sequence that is duplicated such that the polymorphism occurs between the two duplicate loci rather than between two alleles of the same locus. Similarly, variants known as multisite variant (MSV) are found at multiple paralogous sites in duplicated segments, producing polymorphisms due to variation in the number of copies of segments carrying different alleles (Fredman et al., 2004). The mistaken discovery of PSVs and MSVs due to the high proportion of duplicated loci in the rainbow trout genome has greatly limited the conversion and validation of true bi-allelic SNPs from previous discovery projects in rainbow trout (Castano-Sanchez et al., 2009).

In our more recent SNP discovery effort we have demonstrated the effectiveness of using doubled-haploid (DH) homozygous fish in the discovery panel for filtering out likely PSVs (Palti et al., 2014). In DH fish all loci are expected to be homozygous, hence the detection of heterozygous SNPs in the DH lines indicates that the polymorphism likely occurs between two duplicated loci rather than between two alleles of a single locus. For that reason we have also included samples from 11 DH lines from the Washington State University (WSU) collection in our current SNP discovery panel. The WSU DH lines represent populations from the natural rainbow trout distribution in the Pacific Northwest of North America, including the inland subspecies (*O. mykiss gairdneri*) and the coastal subspecies (*O. mykiss irideus*), although most come from hatchery strains at various stages of domestication.

The results of variant calling varies between pipelines, but the agreement between pipelines tends to increase when strong stringency filters are applied to the results (O'Rawe et al., 2013; Li, 2014). In a recent study, Hwang (Hwang et al., 2015) systematically compared several variant calling pipelines and found that *BWA-MEM* (Li, 2013) performed best to map Illumina reads to a reference genome and *SAMtools mpileup* (Li, 2011) did best for variant calling. However, *FreeBayes* (Garrison and Marth, 2012), which employs a haplotype based variant detection strategy, could be a good alternative when only high quality variants (QUAL > 30) are considered (Hwang et al., 2015).

In this study, we used whole genome shotgun resequencing data from DH lines together with aquaculture, hatchery and wild populations to produce a comprehensive SNP dataset for rainbow trout and steelhead. The recently released rainbow trout genome assembly (GenBank assembly Accession GCA_002163495) was used as the reference, and *BWA-MEM* was used to map the sequences to the reference. We employed two pipelines, *SAMtools mpileup* and *FreeBayes*, for variant calling, and filtered the SNPs with a series of stringent filters, including using data from the 11 DH lines to filter out PSVs and MSVs. The SNP database generated will be useful for improving the current whole genome SNP genotyping arrays for rainbow trout as well as for targeted SNP selection in smaller studies that are focused on specific genome regions or particular populations.

## Materials and methods

### Origin of samples and genome sequence coverage

Genomic DNA was extracted from fin clips of 61 rainbow trout. Whole-genome paired-end sequencing libraries were prepared and sequenced using the Illumina HiSeq 2000 or 2500 platforms providing an average of 15X and minimum of 8X genome coverage per sample. The sequenced samples included 11 clonal lines from WSU (Table 1), 38 steelhead and resident rainbow trout from wild and hatchery-origin populations distributed throughout the native range of the species, and 12 fish from the AquaGen rainbow trout aquaculture breeding program. The population of origin and genome sequence coverage of each sample are given in Supplemental File S1.

**Table 1:** List of the 11 clonal doubled haploid rainbow trout and steelhead lines used in this whole-genome resequencing for SNP discovery study.

**Line name**	**Sex**	**Geographic origin**	**Life history type**	**Wild or domesticated origin**
Whale rock male	YY male	Central California Coast	Landlocked steelhead	Wild
Whale rock female	XX female	Central California Coast	Landlocked steelhead	Wild
Arlee	YY male	Northern California	Resident	Domesticated
Hot creek	YY male	Northern California	Resident	Domesticated
Oregon State University	XX female	Northern California	Resident	Domesticated
Golden	YY male	Northern California	Resident	Domesticated
Skookumchuck	YY male	Chehalis River^*^	Winter Steelhead	Semi-wild
Klamath	YY male	Williamson River^†^	Possibly resident	Wild
Skamania	XX male^‡^	Lower Columbia River^*^	Summer steelhead	Semi-wild
Touchet	YY male	Walla Walla River^*^	Inland summer steelhead	Wild
Clearwater	YY male	Snake River^#^	Inland summer steelhead	Semi-wild
Swanson^@^	YY male	Kenai Peninsula, Alaska	Resident	Semi-domesticated^@^

*Information is also provided on the Swanson line, which is the source of the current rainbow trout reference genome*.

* *Washington tributary*.

† *Oregon tributary*.

‡ *This line is phenotypically a male, but it lacking the sdy gene*.

# *Idaho tributary*.

@ *The line was established from a fish that was in the second generation of the hatchery program, or two generations removed from the wild origin of this population*.

### Sequence mapping, variant calling, and SNP filtering

Sequence reads from each sample were mapped to the recently released rainbow trout reference genome (GenBank assembly accession GCA_002163495) using *BWA MEM* (Li, 2013). After the initial alignment, we ran *SAMtools fixmate* to clean up the read pair information and flags, and *SAMtools sort* to sort the alignment data by the chromosome and scaffold locations. Afterwards, PCR duplicates were removed using *SAMtools rmdup*.

After post-processing of the alignment files for the 61 samples, we conducted variant calling using the bioinformatics pipelines of *FreeBayes* (Version 1.0.2) and *SAMtools mpileup*. In both pipelines, we required a minimum mapping quality score (Li et al., 2008) of 30 and a minimum base quality score of 20 for processing a variant site. The variant calling results contained different types of polymorphisms such as SNPs, insertions, deletions, multi-nucleotide polymorphisms (MNP), and other complex events. As the focus of this work was to build a SNP database, we extracted only the SNP variants from the variant calling results and filtered the results to minimize the false positive SNP calls using the following SNP filters:

1. SNP quality filter (QUAL): We only extracted the SNPs that are bi-allelic, not located within 4 bases distance to an indel, and have the phred-scaled variant quality score, QUAL, larger than 30.
2. Low-complexity filter (LC): We removed the SNPs that are in the low-complexity regions in the genome that were identified by the program *mdust* (https://github.com/lh3/mdust/) as suggested by Li (2014). Overall, 1,582,932 low-complexity regions with a total size of 232,458,402 bp, or about 10% of the genome, were identified.
3. Maximum depth filter (DP): We removed the sites that were covered by excess number of reads as these sites are very likely to be located within a repeat or a multi-duplicated region. Although *FreeBayes* and *SAMtools mpileup* report the read depth in slightly different manner due to algorithm differences, we chose the same maximum depth value of 1,500, corresponding to a coverage of 24.6 reads per sample (Li, 2014), for filtering the results from both pipelines.
4. Minimum sample filter (NS): Only SNP sites with sequence reads coverage from at least 58 of the 61 samples passed this filter, to ensure that the majority of the samples were represented in SNP calling for all the sites. To call genotype for an individual sample, we required at least two reads to support the alternate allele and one read to support the reference allele.
5. Double haploid filter (DH): To filter out putative PSVs and MSVs, we employed the same strategy previously used for RAD sequence data (Palti et al., 2014). Briefly, we filtered out SNP sites with heterozygous genotypes in at least two of the 11 DH lines that we re-sequenced in this study.

### SNP validation

To validate a sub-sample of the SNPs with an existing dataset, we identified SNPs from the Affymetrix 57K SNP array (Palti et al., 2015a) that uniquely mapped to exactly the same position in the genome as SNPs developed in the current study. We then used genotype data from the rainbow trout populations previously described by Palti et al. (2015a) to assess the quality of the SNPs from the current study. It is important to note here that the SNPs selected by Palti et al. for the 57K SNP array were pre-filtered for potentially being of higher quality, and hence using this sub-sample of SNPs may be up-biased potentially over-estimating the SNP validation success rate.

### Functional annotation of SNP sites

The putative SNPs were annotated with SnpEff (version 4.3p), an automatic pipeline for rapidly categorizing the effects of SNPs based on the annotation data of a reference genome (Cingolani et al., 2012). The annotation file for the recently released rainbow trout reference genome from NCBI RefSeq (RefSeq assembly accession GCF_002163495.1) was used in building the SnpEff database. The SNPs VCF file was used as the input file, and the results of SnpEff contain the classifications of the effects of the SNPs based on the annotated protein-coding genes and their genomic locations.

### Phylogenetic analysis

A Phylogenetic tree constructed with the SNP data of fish from eight populations that had at least four samples (Table 2). The SNPhylo pipeline (Lee et al., 2014) was used for generating the tree with the program default thresholds. The SNPs used in the analysis were located in the 29 chromosome sequences with MAF > 0.1 and genotype missing rate < 0.1. A linkage disequilibrium (LD) threshold of *r*^2^ > 0.1 was used to reduce SNP redundancy. The tree was built with the maximum likelihood method from the DNAML program in the PHYLIP package (Felsenstein, 1989) and the number at each node represents the percentage bootstrap value determined with 1,000 re-samples using the R package “phangorn” (Schliep, 2011).

**Table 2:** SNP distribution on chromosomes and unplaced scaffolds.

**Chromosome**	**Length (bp)**	**No. of SNPs**	**Average SNP rate (bp)**
Chromosome 1	84,884,017	1,359,811	62
Chromosome 2	85,480,851	1,344,028	63
Chromosome 3	84,937,469	1,228,012	69
Chromosome 4	85,056,421	1,418,468	59
Chromosome 5	92,202,553	1,502,172	61
Chromosome 6	82,930,723	1,223,031	67
Chromosome 7	79,763,776	1,256,322	63
Chromosome 8	83,778,284	1,337,003	62
Chromosome 9	68,467,736	1,111,656	61
Chromosome 10	71,056,191	1,102,388	64
Chromosome 11	80,278,304	1,334,513	60
Chromosome 12	89,655,008	1,323,418	67
Chromosome 13	66,052,243	765,805	86
Chromosome 14	80,358,725	1,123,544	71
Chromosome 15	63,368,167	1,007,882	62
Chromosome 16	70,896,079	1,158,190	61
Chromosome 17	76,527,837	1,167,740	65
Chromosome 18	61,719,220	922,176	66
Chromosome 19	59,576,373	972,098	61
Chromosome 20	41,412,012	729,603	56
Chromosome 21	51,929,587	712,355	72
Chromosome 22	48,550,143	919,605	52
Chromosome 23	49,041,849	830,851	59
Chromosome 24	40,362,479	642,785	62
Chromosome 25	82,601,656	1,326,472	62
Chromosome 26	40,182,520	485,126	82
Chromosome 27	45,316,876	688,717	65
Chromosome 28	40,943,904	679,933	60
Chromosome 29	42,631,536	628,383	67
Unplaced scaffolds	229,020,432	1,139,018	201

## Results and discussion

In this study, we generated a resource dataset of 31,441,105 rainbow trout putative SNPs using whole genome shotgun resequencing for SNP discovery in a panel of 11 DH lines and 50 outbred fish from hatchery and natural populations. The SNPs in our database were called by both the *SAMtools mpileup* and *FreeBayes* variant identification pipelines and passed through stringent QC filters. The distribution of the SNPs on chromosomes, the chromosome sequence lengths, the number of SNPs and the average SNP rate are shown in Table 2. The average SNP rate over all chromosomes was one SNP every 64 bp or 15.6 SNPs per 1 kb. Using VCFtools (Danecek et al., 2011) we calculated that the average genome-wide nucleotide diversity π (Nei and Li, 1979) measured in 20 Kb genomic bins was π = 2.3 × 10^−3^. Compared to other studies of whole-genome resequencing in livestock, the SNP rate revealed in this study is similar to the genome average rate reported for bovine (Daetwyler et al., 2014), but higher than the rate reported for pigs (Choi et al., 2015) and substantially lower than the rates reported for the chicken genome (Kranis et al., 2013) and Pacific oyster genome (Gutierrez et al., 2017). However, it is important to note here that some of the differences between studies may also be technical as the SNP discovery pipeline and QC filtering criteria in those other studies were not identical to this study. Within the rainbow trout chromosome sequences, the average rates were between one SNP every 52 bp (Omy22) and 86 bp (Omy13). In the unplaced scaffolds, the average rate was one SNP every 201 bp. The SNP densities on chromosomes Omy13 and 26 were notably lower than in the other chromosomes. This is consistent with our findings from previous SNP discovery and characterization studies in rainbow trout (Palti et al., 2014, 2015b). For Omy13 and 26, the entire chromosome share high sequence homology with other chromosome arms in the genome as a result of delayed re-diploidization (GCA_002163495.1). As a result of this, a higher proportion of the Omy13 and 26 SNPs were filtered out as potential PSVs after analyzing the genotypes of doubled haploid lines (see further discussion below), which was likely the main reason for the lower density of putative SNPs detected on those two chromosomes.

At the first step of our SNP-calling analysis all sequence reads from each sample were aligned or mapped to the reference genome. A summary of the mapping results for each sample can be found in Supplemental File S1. PCR duplicates from the process of sequencing libraries preparation were found in all 61 samples, and the percentage of PCR duplicates removed ranged from 1.6 to 19.1%. The table also shows that for 60 of the 61 samples over 95% of the reads were mapped to the genome, and only one sample from the Aquagen population had a lower mapping percentage of 84%. The average mapping quality score (Li et al., 2008) was between 33.3 and 38.1. Of the mapped reads, over 97% were paired and the average insert size was between 241 and 659 bp per sample. The average mapping coverage was between 8.6x and 35.9x.

The results of SNP calling by the *SAMtools mpileup* and *FreeBayes* pipelines at each filtering steps are summarized in Table 3. Our assumption based on similar studies was that if a SNP is called by both pipelines, the accuracy of this SNP call should be higher than those only called by one of them. Approximately 80% of the total number of putative SNPs were called by both pipelines at the same site and with the same alternative allele (ALT) in each of the five filtering steps. A small portion of the SNPs that were called by both pipelines had different alternative alleles called by each program probably because of differences between the variant calling algorithms of the two pipelines. *FreeBayes* uses a haplotype based variant calling strategy (Garrison and Marth, 2012) and *SAMtools mpileup* calls variant mainly based on alignments (Li, 2011). However, the percentage of the SNPs called by both pipelines at the same site, but with different alternative alleles, decreased as more SNP discovery filters were applied (Table 3). This suggests that the SNP discovery filters we used improved the agreement between the two variant calling algorithms, which consequently indicates an improvement in the overall SNP calling quality.

**Table 3:** Number of SNPs called by *Samtool mpileup only*, by *FreeBayes only*, by both pipelines at a same site with same alternative allele (*Same site and ALT*), and by both pipelines at a same site with different alternative allele (*Same site different ALT*).

**Filtering step**	***Samtool mpileup only***	***FreeBayes only***	Same site and ALT^*^	***Same site different ALT*^†^**
QUAL	7,265,023	3,580,043	43,220,392 (80%)	7,073 (0.016%)
LC	6,525,505	3,014,395	42,444,371 (82%)	6,083 (0.014%)
DP	6,146,820	4,137,345	39,629,341 (79%)	4,155 (0.010%)
NS	7,190,818	3,054,380	34,120,907 (77%)	2,006 (0.006%)
DH	5,799,376	2,855,191	31,441,105 (78%)	1,205 (0.004%)

*The filtering steps are described in the Methods section*.

* *Percentage is taken out of total combined sets*.

† *Percentage is taken out of total SNPs called by both pipelines at the same sites*.

The 31,441,105 putative SNPs we identified were detected in at least 58 of the 61 rainbow trout samples (i.e., sufficient sequence data was available to elucidate genotypic information from at least 58 samples). This is a fairly stringent criteria compared to other published SNP discovery projects in salmonids (Yáñez et al., 2016). However, due to the good genome coverage from all the samples sequenced in this study, the gain in additional SNPs rapidly diminished with the reduced number of samples (Table 4). Most of the SNPs in the database (27,561,442) were detected in all the 61 samples, and by allowing up to three missing genotypes per SNP, we added a total of 3,879,663 SNPs with little or no impact on the quality of the putative SNPs.

**Table 4:** Number of samples used for SNP discovery, and number of SNPs called by *SAMtools mpileup* and *Freebayes*.

**No. of samples**	**No. of SNPs called by *SAMtools mpileup***	**No. of SNPs called by *FreeBayes***	**No. of SNPs called by both**
61	31,333,183	29,310,209	27,561,442
60	3,768,527	3,100,183	2,733,564
59	1,375,968	1,180,917	792,937
58	764,008	706,192	353,162

*The total number of whole-genome sequenced samples was 61. Up to three missing genotypes per SNP were permitted, but most of the SNPs were discovered without missing genotypes*.

### SNP validation

Overall, 49,155 of QC filtered SNPs in our database mapped to the same sites as SNPs from the 57K array that we have previously characterized and validated (Palti et al., 2015a). Approximately 89% (43,603) of those were high quality and polymorphic SNPs in the populations' polymorphism survey (Palti et al., 2015a), suggesting that ~89% of the variants in the SNP dataset are real SNPs. Detail information on the validation of each marker can be found in Supplemental File S2. However, it is important to note here that the SNPs selected by Palti et al. (Palti et al., 2015a) for the 57K SNP array were pre-filtered for potentially being of higher quality, and hence using this sub-sample of SNPs may be up-biased potentially over-estimating the SNP validation success rate.

### Minor allele frequency

**Figure 1:**
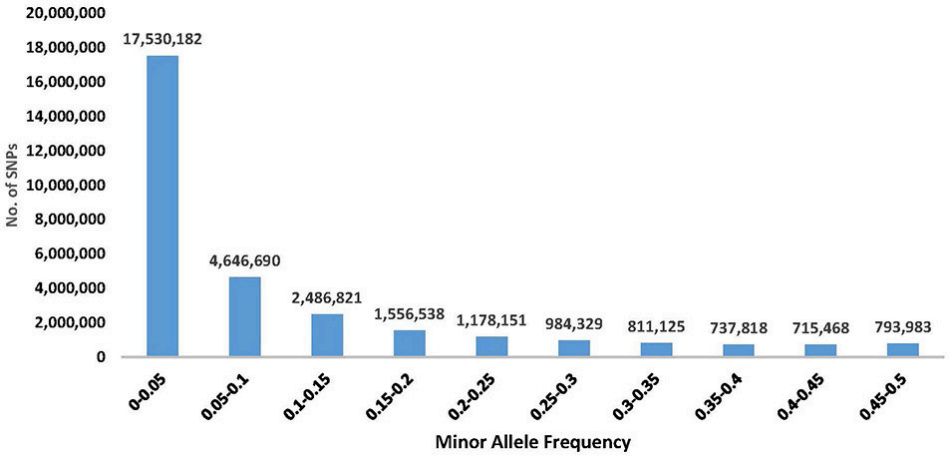
Distribution of SNP minor allele frequency (MAF) in a re-sequencing project of 61 rainbow trout samples (including 11 double haploids). The frequency shown is from a total of 122 alleles per SNP, as each sample was represented by two alleles. Exact number of SNPs for each distribution range category is shown above the corresponding histogram for that range.

The SNPs with higher MAFs are more informative across populations and therefore beneficial on high-density SNP arrays designed for use in multiple studies in a wide range of populations (Palti et al., 2015a). However, low MAF SNPs would typically be specific to only one population and hence potentially more useful for population traceability. In addition, the rare polymorphisms might be associated with unique population traits or with newly emerging phenotypes. The MAF distribution for the SNP database is shown in Figure 1. The number of SNPs with MAF < 0.05 was 17,530,182, which is ~56% of the SNPs in the database. Those low MAF SNP alleles were typically specific to only one population or DH line. On the other hand, there were still a large number of SNPs that were common among most of the samples in this diverse populations' survey, with 4,042,723 SNPs having MAF>0.25. Allele frequency information for each of the 31,441,105 SNPs in the database is provided in Additional File 1.

### Population polymorphism

**Figure 2:**
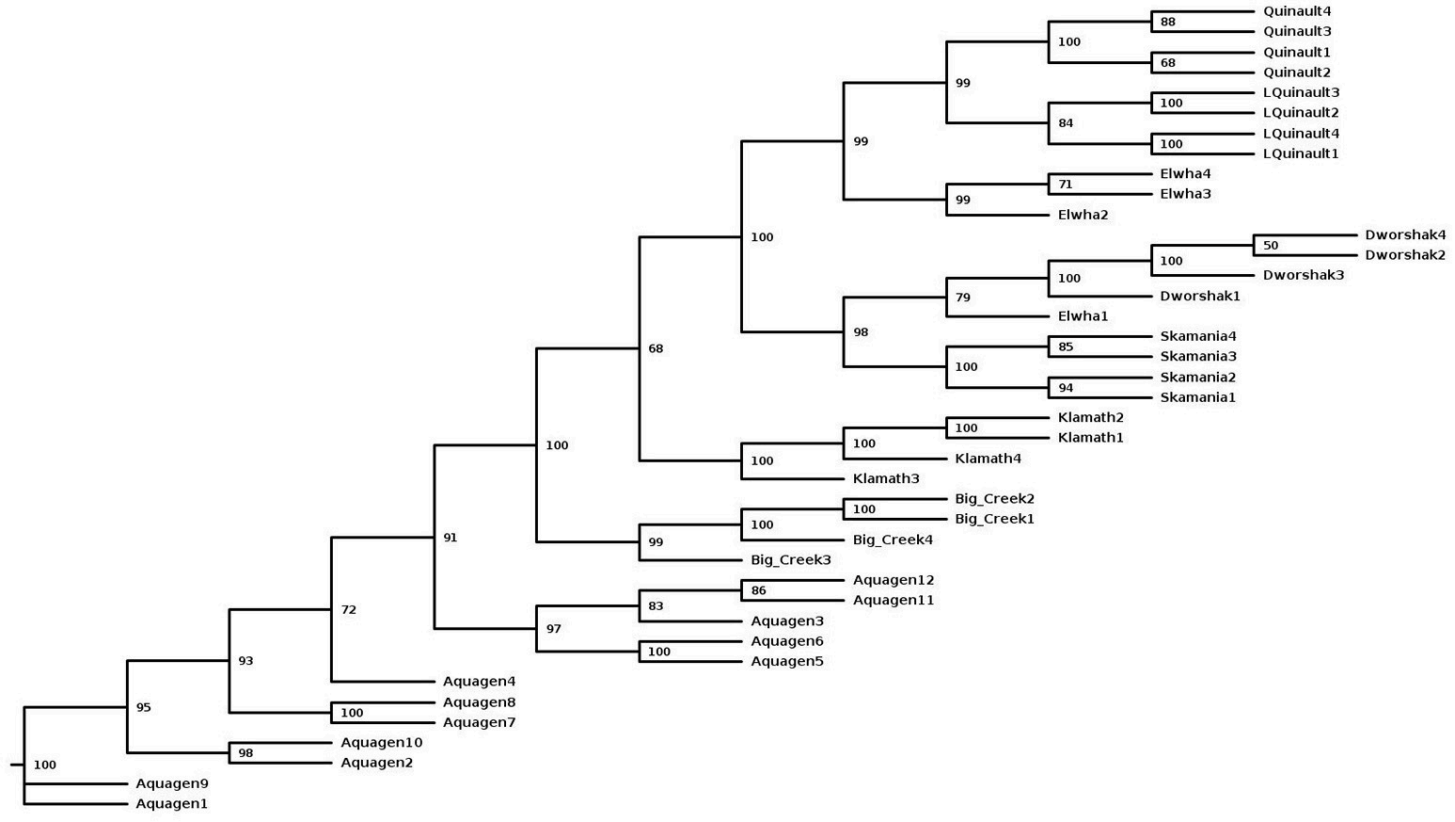
A Maximum-Likelihood phylogenetic tree for 40 rainbow trout sampled from eight populations with a samples size of at least four fish per population. The tree was generated with the SNPhylo pipeline using the program default thresholds for filtering of the SNP genotype data, and using the DNAML program as implemented in the PHYLIP package. The number at each node represents the bootstrap value (percentage out of 1,000 bootstrap samples, estimated with the R package “phangorn”). The population of origin or geographic location is represented in the sample names, following the population nomenclature in Table 5.

To assess the polymorphism of SNPs from the new dataset in individual rainbow trout populations we evaluated the level of marker polymorphism in all the populations from which we have sequenced at least four samples (Table 5). Overall, despite the small sample sizes, our data show large number of polymorphic loci per population and high level of polymorphism and heterozygosity within each population. The number of polymorphic loci per population was between 8.6M (Dworshak) and 11.9M (Aquagen), with 14.4M among the 11 DH lines. For the polymorphic loci in each population, the average MAF was between 0.211 and 0.239, and the average heterozygosity was between 0.316 and 0.400. To asses polymorphism between populations we conducted phylogenetic analysis with fish from same populations (Figure 2). The fish were perfectly grouped by population and/or geographic location of sampling, with the exception of one fish sampled from the Elwha River that clustered with the Dworshak samples. Overall, the phylogenetic analysis results indicate that the SNP database contains enough population-specific polymorphisms to distinguish between the populations used in this study despite the very small sample size used from each population.

**Table 5:** Number and percent of polymorphic SNPs per population or group of samples.

**Population**	Type^*^	**N^†^**	**No. of SNPs^‡^**	**No. Polymorphic**	**% Polymorphic**	**Average MAF^#^**	**Average Het^@^**
Dworshak	H	4	31,356,857	8,642,206	28	0.23	0.40
Quinault	H	4	31,336,970	9,414,415	30	0.23	0.38
L. Quinault	H	4	31,352,692	9,622,312	31	0.24	0.38
Elwha	W	4	31,389,210	11,149,740	36	0.22	0.37
Skamania	H	4	31,346,034	9,408,390	30	0.23	0.37
Big Creek	W	4	31,071,820	11,504,243	37	0.23	0.35
Klamath	W	4	30,742,045	10,469,080	34	0.24	0.35
Aquagen	A	12	30,856,284	11,908,286	39	0.21	0.32
DH Line	DH	11	29,951,350	14,423,126	48	0.19	0.01

* *W, Wild; H, Hatchery; A, Aquaculture; DH, doubled haploid*.

† *Number of fish that were genotyped from that population or group*.

‡ *Number of SNPs with genotype data for all the fish from that population or group*.

# *Average minor allele frequency (MAF) from all the polymorphic SNPs in each population*.

@ *Average observed heterozygosity from all the polymorphic SNPs in each population*.

### Functional SNP annotation

SNPs that can cause gain or loss of function can modify the protein coded by the gene and have major impact on the phenotype of the organism. Therefore, it is important to annotate the SNPs with a potential functional impact in the new SNP database. The functional annotations of the potential SNP effects are listed for each SNP in a supplemental VCF file (Additional File 2) and are summarized in Table 6; including the Sequence Ontology (SO) terms of the SNP effects (http://www.sequenceontology.org/), the potential impact of the single base substitution and the number of SNPs found in each effect type category. Most of the SNP effects (98%) were classified as unknown with the “MODIFIER” putative impact. However, there were 15,198 SNP effects that were considered as HIGH putative impact and 1,579,314 with MODERATE or LOW putative impact. In addition, SnpEff also classified 7,108, 625,238 and 734,274 SNP effects as NONSENSE, as MISSENSE and as SILENT functional classes, respectively.

**Table 6:** Summary of SnpEff annotation with the number of predicted effects in each effect type specified using the sequence ontology (SO) terms.

**Sequence ontology term**	**Putative impact**	**No. of effects**
stop_gained	HIGH	7,108
splice_donor_variant	HIGH	3,068
splice_acceptor_variant	HIGH	2,765
stop_lost	HIGH	1,537
start_lost	HIGH	720
missense_variant	MODERATE	622,938
splice_region_variant	MODERATE or LOW	163,143
synonymous_variant	LOW	733,474
5_prime_UTR_premature_start_codon_gain_variant	LOW	58,871
stop_retained_variant	LOW	796
initiator_codon_variant	LOW	92
non_coding_transcript_variant	MODIFIER	32,353,397
intron_variant	MODIFIER	30,231,828
intergenic_region	MODIFIER	15,397,666
upstream_gene_variant	MODIFIER	12,261,411
downstream_gene_variant	MODIFIER	12,225,390
3_prime_UTR_variant	MODIFIER	1,119,901
intragenic_variant	MODIFIER	400,609
5_prime_UTR_variant	MODIFIER	361,306
non_coding_transcript_exon_variant	MODIFIER	248,075

*Putative impact of each effect is also included in the table*.

### Analysis of DH lines (identification of putative PSVs and MSVs)

To identify putative PSVs and MSVs we analyzed the whole genome resequencing data from the 11 doubled haploid lines, with the assumption that single-locus SNPs are expected to be homozygous in a doubled haploid fish. As previously discussed by Palti et al. (2014), there may be very low occurrence residual maternal loci that were integrated into the genomes of the otherwise doubled paternal haploid genomes, and therefore it is safer to use a threshold of heterozygous in at least two DH lines (Het > 1) for identifying putative PSVs and MSVs. Most of the PSVs and MSVs are thought to represent duplicated loci, although they can also be caused by loci that occur in more than two copies in the genome. In the last step of our SNP discovery filtering QC we removed all the putative PSVs and MSVs that were found to be heterozygous in two or more DH lines, because those loci would often fail to genotype using common commercial genotyping platforms designed for diploid loci. However, duplicated loci may be proven useful for studying adaptation in salmonids (Limborg et al., 2016) and may also improve accuracy of population assignment (Gilbey et al., 2006). For that reason, we included in Additional File 3 a database of all the putative PSVs and MSVs that were filtered out from our SNPs database, but may be useful for investigations that are focused on duplicated regions and loci in the rainbow trout genome.

**Figure 3:**
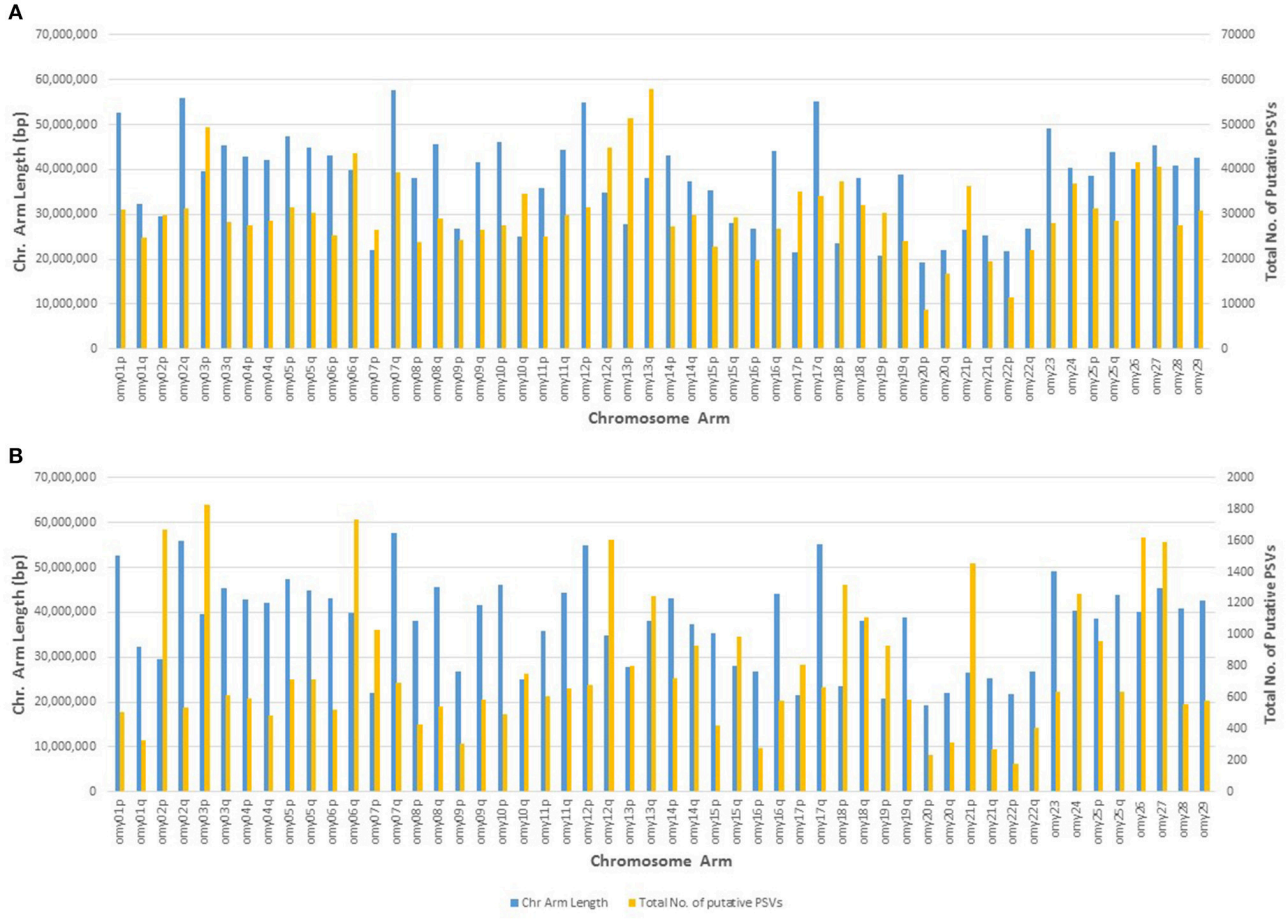
Genomic distribution of putative PSVs from the analysis of resequencing data from doubled haploid lines. The number of putative PSVs and MSVs on each chromosome arm is plotted against the chromosome arm length in base pairs. Putative PSVs and MSVs were counted as SNPs that were heterozygous in at least two DH lines (Het > 1) **(A)**, or Het in all 11 lines **(B)**. The 14 chromosome arms with delayed re-diploidization in the rainbow trout genome are 2p, 3p, 6q, 7p, 10q, 12q, 13p, 13q, 15q, 17p, 18p, 19p, 21p, and 26. Those chromosome arms have much higher density of putative PSVs and MSVs compared to the rest of the chromosome arms in the rainbow trout genome.

The genomic distribution of the putative PSVs and MSVs on chromosome arms is plotted against the length of each chromosome arm in Figure 3. In rainbow trout there are seven pairs of chromosome arms which display delayed rediploidization with clusters of very high sequence similarity, including arms 2p-3p, 6q-26, 7p-18p, 10q-19p, 12q-13q, 13p-17p, and 15q-21p (Lien et al., 2016). Therefore, we expect to find higher frequency of PSVs or MSVs on those 14 chromosome arms; and indeed as we show in Figure 3A, the ratio between the numbers of putative PSVs and MSVs we identified in the DH lines and the chromosome arms' length in base-pairs is much higher among those 14 chromosome arms compared to the rest of the genome. Furthermore, when we increase the threshold to only loci that are heterozygous in all 11 DH lines (Figure 3B), we can see a further increase in the overrepresentation of the putative PSVs and MSVs among the 14 duplicated chromosome arms. The draw-back of increasing the threshold is an overall reduction of the number of loci that can be detected. As shown in Table 7, when we used a threshold of Het > 1 among the 11 DH lines we identified ~1.7M putative PSVs/MSVs with an average locus observed heterozygosity of 0.44 and with 39% of the loci not in Hardy-Weinberg equilibrium (*P* < 0.05), among the 50 outbred or non-DH rainbow trout that we sequenced. In comparison, for the ~31.4M SNPs that passed our filtering criteria among the same set of 50 fish, only 12% of the loci were not in Hardy-Weinberg equilibrium (*P* < 0.05) and the average observed heterozygosity was 0.12, indicating excess heterozygosity among many of the ~1.7M putative PSV/MSV loci that were filtered out of the SNP database. Higher average observed heterozygosity than expected per locus is a strong indicator for potential MSVs or PSVs among the surveyed loci (Hohenlohe et al., 2011; Lien et al., 2011). As expected, with a threshold of Het = 11 among the DH line, the average observed heterozygosity among the 50 non-DH fish increased to 0.85 with 95% of the loci not in Hardy-Weinberg equilibrium. However, the number of putative PSV/MSV loci identified dropped dramatically to only 40,697, indicating that the majority of the true PSV/MSV loci would be missed and not be filtered out with this very stringent threshold.

**Table 7:** The effect of threshold of minimum number of heterozygous genotypes (Het) among the 11 doubled haploid (DH) lines on the number of putative PSVs and MSVs identified and the average observed heterozygosity in those loci among the 50 outbred (non-DH) rainbow trout sampled in this study.

**No. of het genotypes**	**No. of putative PSVs/MSVs**	Average heterozygosity^*^	**Deviation from HW (%)^†^**
Het > 0	2,767,612	0.35	28
Het > 1	1,733,481	0.44	39
Het > 2	1,187,715	0.51	49
Het > 3	836,278	0.57	58
Het > 4	596,329	0.62	67
Het > 5	429,862	0.66	75
Het > 6	311,629	0.71	81
Het > 7	224,252	0.75	85
Het > 8	155,617	0.78	89
Het > 9	97,433	0.82	92
Het = 11	45,757	0.85	95

*High rate of observed heterozygosity indicates high occurrence of MSVs and PSVs*.

* *Average observed heterozygosity per locus*.

† *Percent of loci that deviated from expected Hardy-Weinberg equilibrium (P < 0.05)*.

## Conclusion

Using the new rainbow trout reference genome (GenBank assembly Accession GCA_002163495), and genome resequencing of 61 rainbow trout and steelhead samples, including 11 doubled haploid clonal lines, we discovered 31,441,105 high quality SNP loci. The SNP loci are broadly distributed across the 29 chromosomes with a genome-wide average rate of 15.6 SNPs per 1 kb. The new SNP database, together with their functional annotation, provide an important resource for new SNP array designs, whole genome association analyses, and design of genotyping assays in rainbow trout.

## Accession number

Raw sequence data that were generated in this study were deposited in the NCBI short reads archive under accession SRP107028. (Project ID: PRJNA386519).

## Data accessibility

DNA sequences: NCBI SRA accession SRP107028 (BioProject ID: PRJNA386519).

The VCF file for the database of all the SNPs identified in this study including the SnpEff annotation information and a file with allele frequency information for each SNP in the database are available for downloading from a public repository (AnimalGenome.org).

## Author contributions

GG: performed bioinformatics and data analysis and wrote the manuscript draft; TN: assisted with the study design and data collection and contributed to the data analysis; DP: co-designed the study and contributed samples; TM: collected samples and contributed DNA sequence data; KN: collected samples and contributed sequence data; GT: co-designed the study, collected samples and contributed sequence data; SL: co-designed the study and co-directed the work; YP: co-designed the study and directed the work and contributed to the data analysis and first draft writing. All authors reviewed the manuscript draft and approved its content.

### Conflict of interest statement

The handling Editor declared a past co-authorship with several of the authors KN, SL, and YP. TM was employed by company AquaGen. The other authors declare that the research was conducted in the absence of any commercial or financial relationships that could be construed as a potential conflict of interest.

## Supplementary material

Supplementary File S1 Actual sequence coverage and genome mapping information for each of the 61 samples.

Supplementary File S2 Validation of the putative SNPs using the 57K SNP chip data.

Available for downloading from https://www.animalgenome.org/repository/pub/USDA2018.0209/

Additional File 1 Allele frequency information for each of the 31,441,105 SNPs in the database.

Additional File 2 A VCF file generated by the program mpileup for the database of all 31,441,105 SNPs identified in this study including the SnpEff annotation information for each SNP.

Additional File 3 A database of all the putative PSVs and MSVs that were filtered out from our SNP database, including location in the reference genome and alternative alleles.
